# Perceptions of building-integrated nature-based solutions by suppliers versus consumers in Egypt

**DOI:** 10.1038/s41598-024-76014-8

**Published:** 2024-10-30

**Authors:** Mai A. Marzouk, Mohamed A. Salheen, Leonie K. Fischer

**Affiliations:** 1 Institute of Landscape Planning and Ecology ILPÖ, Faculty of Architecture and Urban Planning, University of Stuttgart, Stuttgart, Germany; 2 Department of Architecture, Faculty of Engineering, Ain Shams University, Cairo, Egypt; 3 Department of Urban Design and Planning, Faculty of Engineering, Ain Shams University, Cairo, Egypt; 4 Integrated Urbanism and Sustainable Design (IUSD) Program, Ain Shams University, Cairo, Egypt

**Keywords:** Sustainability, Socioeconomic scenarios, Climate-change mitigation, Psychology and behaviour, Climate-change adaptation

## Abstract

Can Building-Integrated Nature-based Solutions (BI-NbS) reach their full potential in the Global South? In the Egyptian context, BI-NbS are relatively new with an identified gap between the high potential in theory and low implementation rates in practice. To bridge this gap, the study conducts an in-depth investigation of BI-NbS market conditions to reveal the current trends in the residential buildings market in Egypt. It also identifies the gaps and overlaps in the perceptions of the suppliers and consumers of BI-NbS. Results reveal that the residential sector sales mainly target high-income groups yet very limited and dominated by rooftop systems. Suppliers advocate for high-tech systems over low-tech systems, whereas consumers prefer the latter. The perceptions of suppliers and consumers mostly align regarding the basic aspects such as the production and operation preferences as well as the anxieties and concerns about the relatively new BI-NbS in this regional context. However, they diverge in key aspects affecting market penetration such as implementation conditions, aims, and barriers. Accordingly, the study identified the gap between suppliers and consumers, and outlined recommendations, directed to suppliers and policymakers, for improved market development and local implementation of BI-NbS in emerging markets of the Global South, such as Egypt.

## Introduction

Cities of the Global South are facing several societal challenges, relating to climate change, food security, and human well-being^1–3^, which are intensified due to rapid urbanization and economic hurdles^4–6^. Focusing on Egypt, several of these challenges have intensified in the past years, and by 2050, the Egyptian population is expected to exceed 160 M, with the urban population surpassing the rural one and reaching 55%^7,8^. The resulting massive expansion of urban areas imposes multiple challenges that are worsened by global warming^9^. These include an annual loss of 300 Km^2^ of agricultural land and a projected 6% loss in food production by 2050^10,11^. Such implications jeopardize the well-being of urban residents, whose physical health is affected by malnutrition, 28.5% of the population suffers from moderate/severe food insecurity, and 13% from stunting^12,13^. In addition, in 2022, food inflation rates reached 37.8%, forcing households to shift to more affordable unhealthy diets^14^. Not only physical but also mental health is affected, especially by the limited access to green spaces^15,16^, which is estimated at 0.74–3 m^2^ per capita in Cairo for example^17,18^, compared to the international standard of 12–18 m^2^^19,20^. In fact, research proved a strong association between green spaces and positive mental health outcomes^21–23^.

**Fig. 1:**
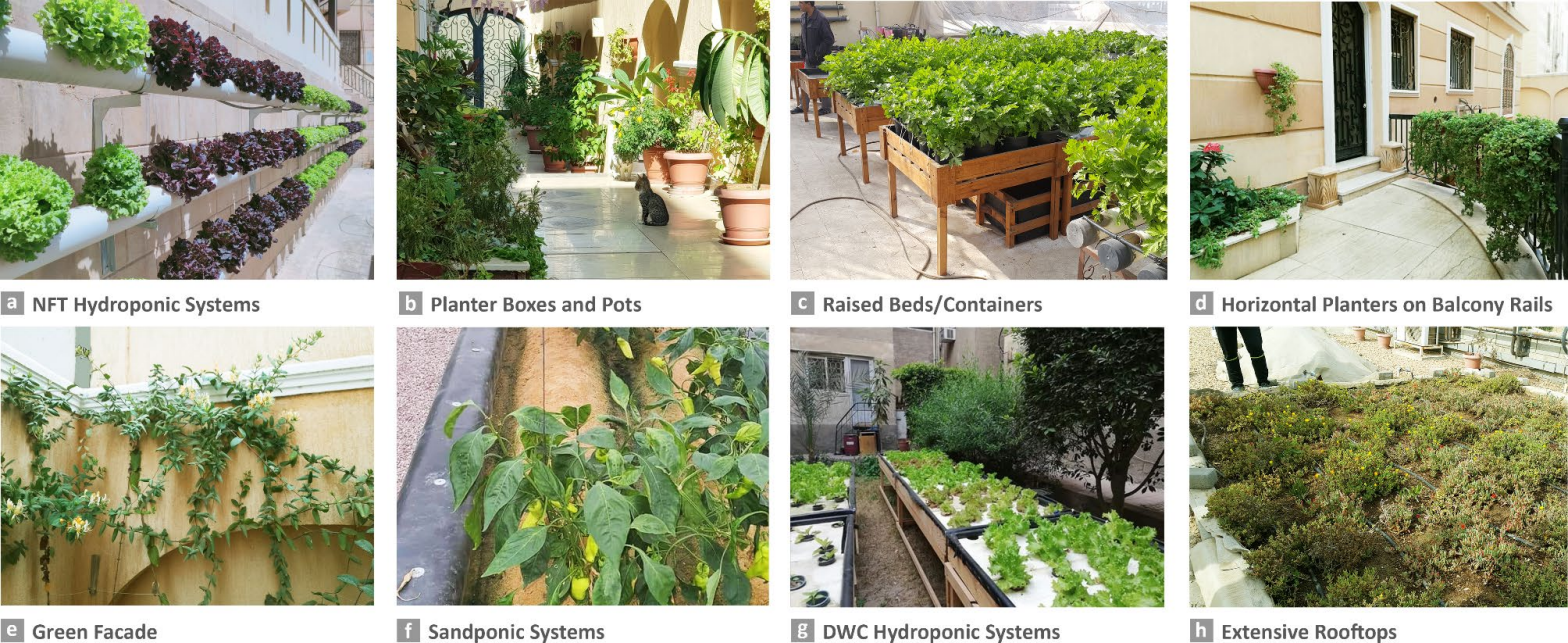
Building-Integrated Nature-based Solutions (BI-NbS) covered in the study. The panel shows the systems that achieved the highest sales in the Egyptian market according to market players ^42^. Credits of (**a**, **f**–**g**): El Zain, and (**b**–**e**, **h**): Mai Marzouk.

Nature-based Solutions (NbS), defined by the European Commission (EC) in 2015^23^, have risen on the international agenda. They represent a promising approach to addressing these societal challenges by integrating natural elements and processes into urban areas to improve the environmental, ecological, social, and economic conditions^24,25^. NbS address seven societal challenges as identified by IUCN including climate change adaptation and mitigation (SCh1), disaster risk reduction (SCh2), socioeconomic development (SCh3), human health (SCh4), food security (SCh5), water security (SCh6), and ecosystem degradation and biodiversity loss (SCh7)^2,3^. Furthermore, the EC identified "enhancing sustainable urbanization" as the first goal of NbS^23^, and emphasized the need to investigate the effectiveness of pre-existing and new, greening and agricultural systems as NbS, under its research and innovation actions^23^. In line with this, our study focuses on pre-existing systems in the Egyptian market that qualify as NbS at the building scale^3^, which could contribute to this goal^23^ and address several of the outlined societal challenges^2,3^. For our study, these include greening systems, such as green roofs and vertical greening systems^26–28^, which serve as alternative green spaces by incorporating diverse and decorative plants. In correspondence to the IUCN challenges, these achieve multiple social and environmental benefits such as nature exposure (SCh4), aesthetic view enjoyment (SCh3&4), and air quality improvement (SCh1&4)^29–33^. Additionally, agricultural systems like rooftop agriculture and vertical farms, which produce edible plants^34,35^, are included under the umbrella of NbS^6,36,37^ due to their numerous health and environmental benefits, including healthier food provision (SCh4), food self-sufficiency (SCh5), and microclimate improvement (SCh1)^33,38,39^. Thus, our study uses the term “Building-Integrated NbS (BI-NbS)” to refer to type 3 NbS, which involves the design and management of new ecosystems, such as artificial ones (e.g. green buildings)^1–3,40,41^. BI-NbS are implemented on building envelopes (rooftops, façades, and balconies) to grow both decorative and edible plants (Fig. 1).

NbS theory is not yet fully reflected in their practice, due to knowledge gaps and missing partnerships between the stakeholders involved in implementation^6,26^. In addition, most research and practice of NbS is biased towards the Global North with a limited understanding of the dynamics of the Global South despite its higher vulnerability to societal challenges^4,5^. This conclusion was supported by the global NbS mapping conducted by Goodwin et al.^4^, which revealed the need for filling the gaps and transferring lessons learned from the north to the south to ensure the effectiveness of NbS in different contexts. The researchers also pinpointed the need for stronger collaboration between science and policy to comprehend how contextual dynamics support or hinder the implementation of certain NbS types. To contribute to filling the gap in research on the Global South, Lakshmisha et al.^5^ mapped 120 NbS cases in the Global South, defined their stakeholders, and assessed the adopted participatory approaches. The predominance of blue-green infrastructure systems in the cases was highlighted, which ensures both environmental and social benefits. Nassary et al.^6^, again, reported the need for more studies that illustrate NbS potential to provide numerous benefits to communities in the Global South. Using a systematic review, they showed the outcome of several studies considering urban green infrastructure and urban agriculture as elements of NbS. Additionally, in a study tackling NbS potential in the MENA region, Ben Hassen and Hageer^43^ outlined the different approaches and benefits of NbS for the region as well as the challenges facing them such as the economic barriers, lack of public knowledge, and lack of coordination among stakeholders. They concluded that future studies need to focus on understanding the social components of NbS, including the community perceptions and their engagement tactics, which are crucial for a successful NbS implementation in the region.

For Egypt specifically, NbS literature is mainly centered around interventions used for wastewater treatment, sea level rise adaptations, flood resilience, etc. Only a few studies focused on greening and agricultural systems implemented on the building scale under the umbrella of NbS, such as a study on life cycle assessments of vertical farming as a nature-based solution^44^, or a study on green roofs in Cairo as part of nature-based green infrastructure in Africa^45^. Therefore, it seems crucial to study the variety of selected systems jointly and not independently, and also to include the social aspects and the perceptions of different stakeholder groups, in response to the identified research gaps.

In recent literature on social perceptions of the selected BI-NbS systems, we identified two groups of studies: (1) Professionals’ perceptions studies, that tackle the perceptions of professionals such as architects and developers about the aims and barriers of the systems. Most studies focused on Asian^46–50^ and European contexts^51–54^, and one study tackled the African context^27^. Despite the range of involved professional stakeholders, suppliers were not included in any of these studies. (2) Willingness to Pay (WTP) studies, that measure the consumers’ willingness to pay for such a system mostly in their residential homes. Most studies focused on greening systems in Asian^55–58^, or European contexts^59–61^. To the best of our knowledge, we could not identify comprehensive literature addressing the perceptions of professionals about the selected BI-NbS in the residential sector of Egypt. Interestingly, Elhady et al.^62^ focused on the users’ and experts’ views but in commercial and office buildings, while Fasial et al.^63^ briefly covered the views of different stakeholders about green roofs only. Also, there were hardly any studies that attempted to identify the perceptions of the suppliers versus consumers, that is, comparing the two stakeholder groups. Accordingly, the market conditions of BI-NbS in Egypt remain as an unexplored black box. As a result, the actual implementation of the selected BI-NbS is still lagging in Egypt^63–65^, despite the systems’ outlined benefits, their existence in the Egyptian market^42^, and their social acceptance by end users^66^. To bridge this identified gap, the market conditions in which BI-NbS implementation takes place need in-depth investigation.

Our overall aim is thus to provide an insight into the relatively new market of BI-NbS, integrated into residential buildings, in the untackled context of Egypt, to contribute to the missing links of this research field to the Global South. Our study was divided into two parts, whereas the first part aims to identify the market trends from the perspective of the suppliers. The second part replies to the question of how much the suppliers comprehend the motives of consumers for accepting and implementing the systems. Accordingly, the study at hand defines the perceptions of suppliers versus consumers about BI-NbS acceptance dynamics. In line with that, two research questions were outlined: (1) What are the market trends for BI-NbS in the residential building sector in Egypt? and (2) What are the social acceptance dynamics for BI-NbS from the perception of suppliers (supply) versus consumers (demand)? The novelty of the study lies in going beyond listing the types of NbS, their benefits, and implemented case studies, to explore the market trends and dynamics of BI-NbS from a socioeconomic perspective in an understudied context. This aligns with the current efforts to deepen the understanding of NbS potential from local perspectives to ensure their broader implementation in diverse contexts. The recommendations outlined in this study could help suppliers achieve effective marketing of the systems to consumers, thereby increasing their implementation in urban Egypt. The insights extend beyond Egypt, as an emerging BI-NbS market, to other Global South countries of similar climates, socioeconomic and market conditions, and societal challenges.

## Methods

In this study, two surveys were used to collect data, i.e. from suppliers and consumers of BI-NbS. Like this, data was collected using a first survey directed towards suppliers and experts of BI-NbS (“Suppliers survey”). The SurveyMonkey platform was used to develop English and Arabic online versions of the survey, which was pre-tested with a small sample (n = 3), resulting in a simplified structure and word choice of the final survey. The Suppliers survey had a qualitative nature with a high interest in each participant’s input and contribution to the research. As a result, purposive sampling was used in its dissemination and in the selection of participants to represent the most relevant and influential market players in the sample^61,67^. Participants were selected from a list of specialized suppliers, identified through desktop research and snow-balling techniques^68^. Abiding by COVID-19 regulations at the time of conducting the research, the 10 min survey was shared online over 8 weeks starting in March 2022, using public and/or private social media platforms until reaching the point of data saturation from the collected responses. Finally, 15 suppliers participated out of 20 contacted (i.e., a response rate of 75%). In parallel, the second survey was developed and distributed to end users (“Consumers survey”), specifically residents in new cities of the Greater Cairo Region (GCR) in Egypt. This survey had a quantitative nature and yielded 487 eligible respondents, of which we included 274 complete datasets in the following analyses. Some aspects of the Consumers survey were discussed in previous studies^66,69^, but aspects related to NbS implementation are presented in detail in the study at hand. All methods were carried out following relevant guidelines and regulations. For both surveys, mandatory consent was requested at the beginning and respondents were informed that participation is voluntary, responses are anonymous, confidential, and used solely for research purposes. The surveys were deemed suitable for sharing with the targeted population by the “Ethics committee” at the University of Stuttgart, whereas formal approval was not required as the surveys do not touch upon personal aspects or assess them.

**Fig. 2:**
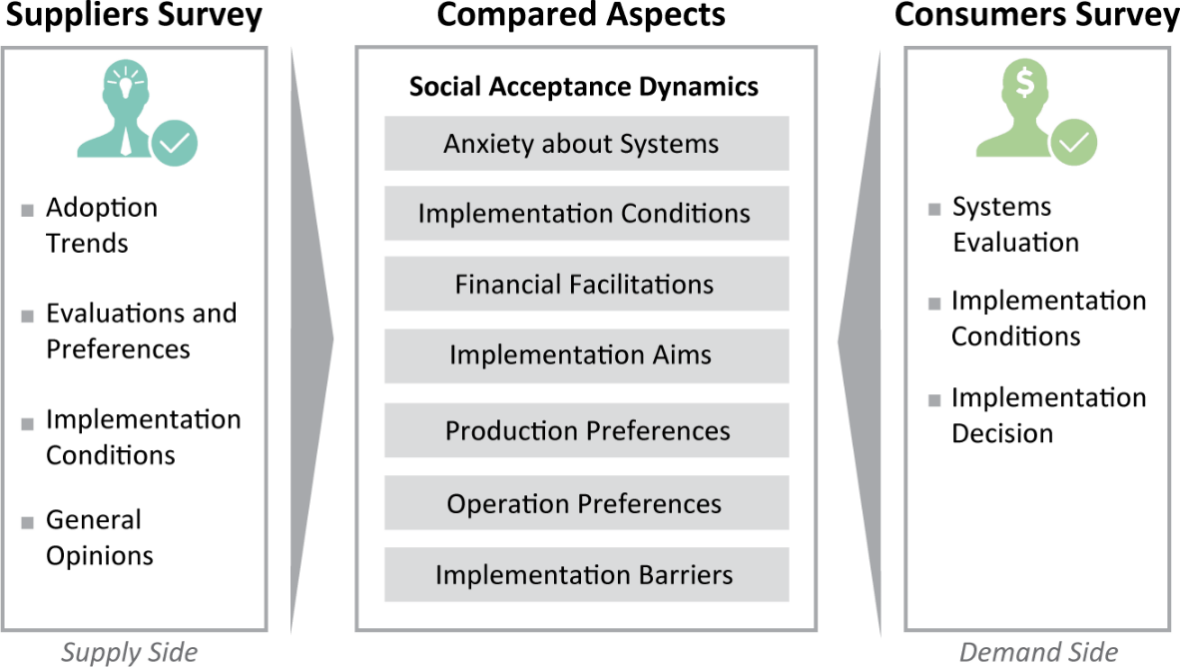
Outline of studied aspects. The social acceptance dynamics covered seven aspects identified from the Suppliers survey (supply side) and the Consumers survey (demand side).

Firstly, we report on data from the Suppliers survey, which assesses the **market trends of BI-NbS **including the following aspects: “residential sector sales”, “BI-NbS typologies sales”, “market segments”, “feasibility of BI-NbS aims”, and “BI-NbS costs and productivity”. Secondly, we report on data from the Suppliers survey, which is mirrored in the Consumers survey to identify the **social acceptance dynamics** in the local context. Seven aspects were discussed from this database, namely “Anxiety about Systems”, “Implementation Conditions”, “Financial Facilitations”, “Implementation Aims”, “Production Preferences”, “Operation Preferences”, and “Implementation Barriers” (Fig. 2), where suppliers were asked about their perceptions of the consumers’ views and preferences. The seven aspects were selected based on the theoretical frameworks developed in previous studies^66,69^, while focusing on the aspects relevant to both stakeholder groups of BI-NbS—suppliers and consumers—to put them into comparison. These aspects revealed novel results in relation to the consumers’ acceptance of the systems, thus they were worth investigating to reveal the extent of their comprehension by the suppliers for a better understanding of the BI-NbS market dynamics. Original questions and response items of both surveys are provided in Supplementary Table S1 and Table S2.

At this early stage of the systems’ local availability, emphasis was given to the in-depth identification of existing gaps and overlaps between the two groups, that is, rather qualitatively than quantitatively. Accordingly, nominal multiple response questions formed the core of the Suppliers survey with some exceptions such as (1) the ordinal rating scale question identifying the residential sector sales, (2) the dichotomous yes/no question covering the feasibility of BI-NbS aims, and (3) the open-ended questions asking about the BI-NbS costs and productivity. Questions tackling the “Anxiety about Systems”, “Implementation Conditions”, and “Financial Facilitations” were based on the Unified Theory of Acceptance and Use of Technology (UTAUT) theory with response items influenced by several studies^70–80^, while questions about “Implementation Aims”, “Production Preferences”, “Operation Preferences”, and “Implementation Barrier” were based on the discussions of previous case studies^47,79,81,82^ and on the outcome of a preliminary analysis of the studied context and systems using semi-structured interviews with key market players. Collected data was processed using the SurveyMonkey platform, and the descriptive analysis was conducted using Microsoft Excel (V 2310) and SPSS (V 25) for both surveys.

## Results and discussion

### Market trends of BI-NbS

To identify the level of market development, suppliers reported on multiple facets of their companies’ sales. First, in regard to **sales by sector**, the average sales of BI-NbS in the residential building sector were 33% of the total sales of surveyed companies, that is, it was likely exceeded by sales to commercial and office buildings or large-scale commercial greenhouses on the ground. This finding is consistent with an opinion highlighted in the preliminary semi-structured interviews with suppliers of the systems.

Second, **sales by BI-NbS typologies**, rooftop systems achieved the highest sales, selected by the majority of respondents (75%), followed by balcony systems (33%), while the façade systems were not selected, indicating the limited implementation of vertical systems in the context^42,83^. This aligns with previous discussions on the popularity of the more developed rooftop systems in other regions^60,61,84,85^. One possible explanation for the lower sales of balcony systems, despite their preference by consumers^66,86^, is that the selected systems were mostly low-tech or Do-It-Yourself (DIY) options^42^. These are often bought in stores and installed without specialized suppliers, hence their limited reflection in sales.

Third, regarding **sales by market segment**, the majority of respondents (67%) indicated that high-income individuals had the highest BI-NbS implementation rates, followed by middle-income (44%) and low-income individuals (22%). The inferred relation between income level and implementation rates is supported by other studies, where willingness to pay, as an indicator of implementation, was considered directly proportional to income level^55,59,61^. One reason could be that higher implementation, especially in new markets, is usually attributed to better knowledge of systems^61,83^ and higher financial capacity, which is common among the affluent groups of society, characterized by higher income, educational, and environmental awareness levels^57,58,69,87^. Nevertheless, the sales distribution among the income groups reflects the diverse opportunities available for BI-NbS types identified in the market. The systems seem to be considered suitable for different groups whether underprivileged or privileged^42^, and for different purposes such as food security (SCh5), community engagement (SCh3), healthy food provision (SCh4), or improved aesthetics (SCh3&4)^3,88^, which links to the seven societal challenges outlined by IUCN^2,3^ (see *Introduction*).

The suppliers also expressed their opinion of the **feasibility of BI-NbS aims**. On the one hand, selling the produce for income generation was considered of low feasibility, whether they are decorative plants (considered feasible by 50%) or edible plants (33%). This is likely attributed to the limited space available in residential buildings, which hinders large-scale production and commercialization of BI-NbS^42,46^. On the other hand, self-consumption of edible plants (83%) and food expenses reduction (83%) were considered equally feasible by the suppliers. However, these benefits were not equally attractive to the consumers, where the former benefit considerably outweighed the latter in their preferences (see *Implementation aims*). Still, the importance of both benefits for the local context was highlighted in previous studies^79,89^.

**Fig. 3:**
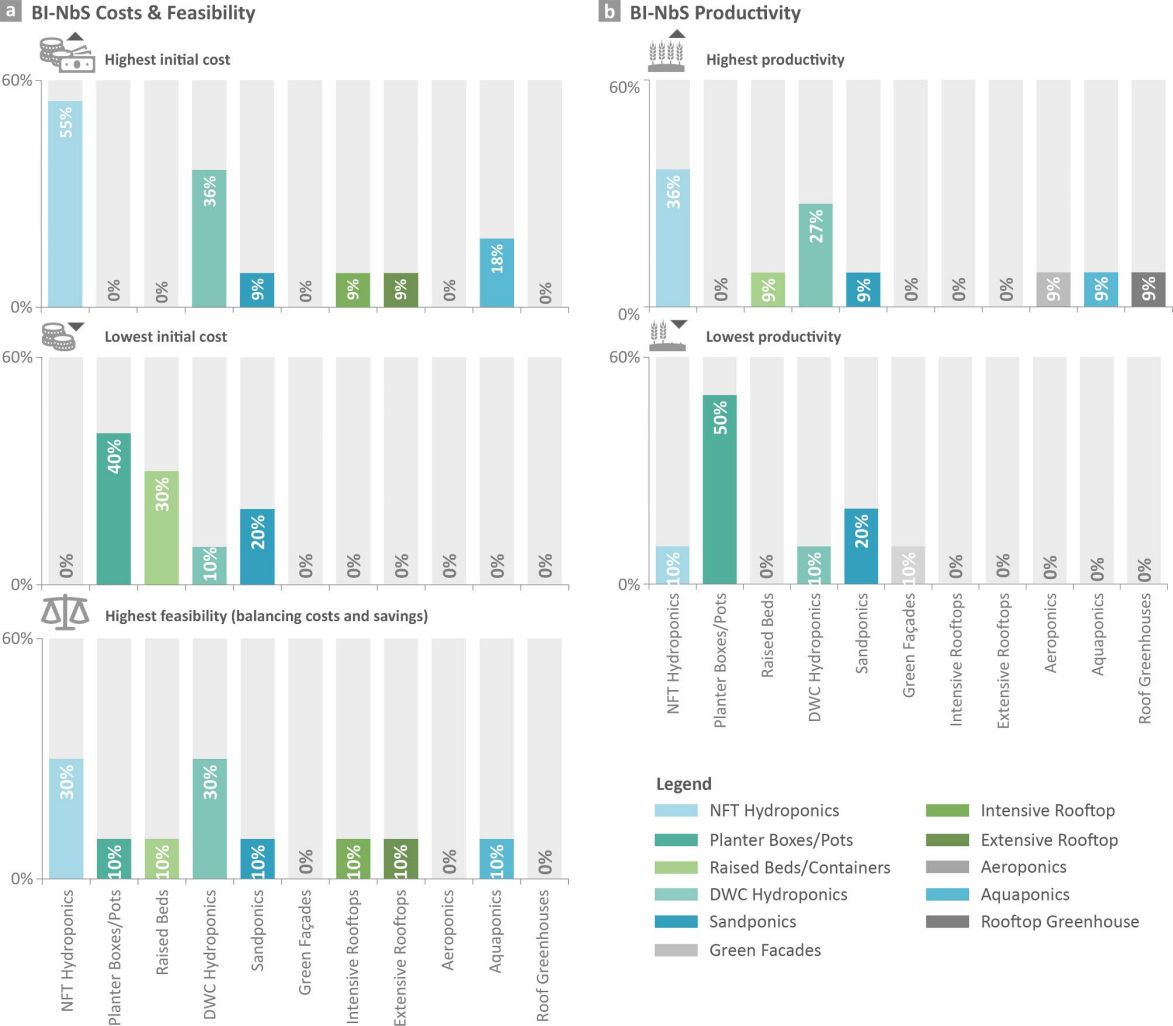
Results of BI-NbS costs and productivity. Respondents were asked to indicate the systems with the highest and lowest costs and productivity, and highest feasibility. The order used in the charts was based on a list of systems that achieved the highest sales in the market ^42^, and the systems not selected in any of the 5 themes by respondents were removed from that list. *NFT:* Nutrient Film Technique, *DWC:* Deep Water Culture.

In regard to **BI-NbS costs and productivity**, Nutrient Film Technique (NFT) and Deep Water Culture (DWC) hydroponics were considered to have the highest initial costs (55% and 36% respectively) and to achieve the highest productivity (Fig. 3; 36% and 27%, respectively). Their high costs were supported by some studies^35,90^, while refuted by others claiming their low initial costs unless automation systems were used^91,92^. Furthermore, their high productivity was attributed to producing more harvest by shortening the growing period of crops (i.e. more cycles annually)^90,91^. On the contrary, planter boxes/pots, raised beds, and sandponics were considered to have the lowest costs in our study (ranging from 20 to 40%), while only planter boxes/pots and sandponics were considered at the lower end of productivity (50% and 20% respectively). Thus, our assessment reveals the high potential of raised-bed systems given their lower cost, owing to low-cost components^88,91^, and reasonable productivity according to the suppliers. Regarding the **BI-NbS feasibility**, NFT and DWC hydroponics achieved the highest rank (Fig. 3). For these, respondents claimed high productivity, which is probably expected to provide enough produce that achieve reasonable savings to balance the high initial costs.

### BI-NbS social acceptance dynamics between supply and demand

#### Anxiety about the systems

**Fig. 4:**
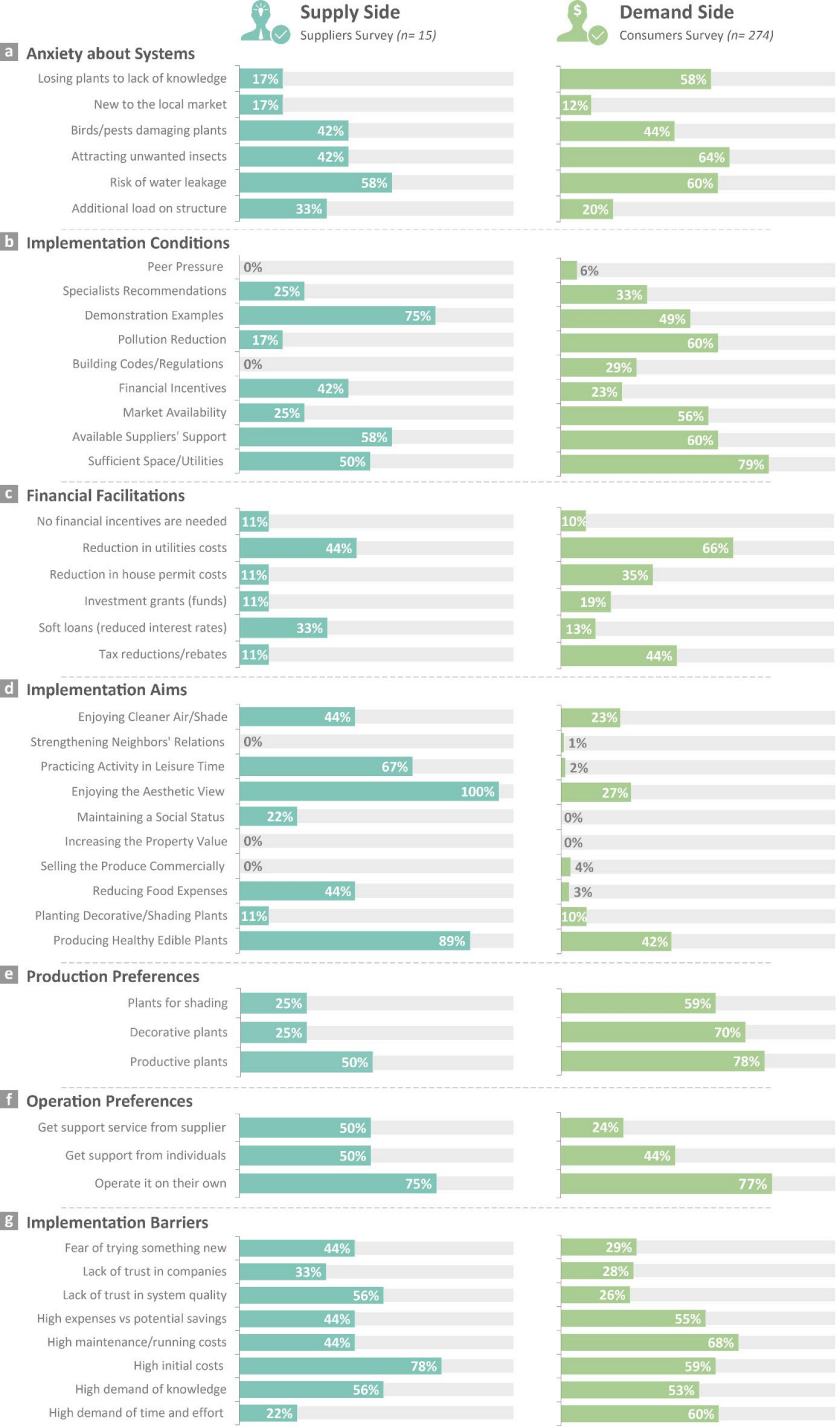
Results of the social acceptance dynamics from the perception of suppliers (n = 15) versus consumers (n = 274), showing the aspects: Anxiety about systems, Implementation conditions, Financial facilitations, Implementation aims, Production preferences, Operation preferences, and Implementation barriers. For more background information on the responses of consumers, please see ^66,69^.

The perceptions identified from the Suppliers and Consumers surveys regarding anxieties about BI-NbS mostly align (Fig. 4a). The fear of water leakage and the fear of plants’ damage by birds/pests, previously depicted in literature^58,93^, were almost equally selected by both groups, highlighting a good understanding of the suppliers for some of the highly reported anxieties by consumers. However, the suppliers underestimated the fear of attracting unwanted insects, the top anxiety for consumers, as supported by studies from Egypt^63,94^ and other countries^56,95^. Also, the fear of losing plants due to lack of knowledge was more intense for consumers than perceived by suppliers. Due to the long tradition of agricultural practice for Egyptians in the so-called kitchen gardens^96,97^, we assume that suppliers considered gardening to be widely known, hence their underestimation of the consumers’ knowledge worries. Conversely, suppliers overestimated the consumers’ worry about the additional load on the building structure, probably driven by their understanding of the importance of system weight and structural load for a successful installation^46,53^.

#### Implementation conditions

We identified some discrepancies in the understanding of suppliers for the most important conditions behind the acceptance of consumers (Fig. 4b). For instance, suppliers underestimated how important it was for consumers to have sufficient space/utilities. This links to the discussion on wasted spaces in residential buildings in Egypt, including cluttered roofs and balconies^80,89^, which could induce a misbelief that there is not enough space for BI-NbS. Therefore, this highlights the necessity of raising awareness on the spatial requirements of BI-NbS, and on the options for using available spaces, even at small scales. Even though demonstration examples were considered important by consumers for increasing their trust in the systems^69^, this was slightly overrated by suppliers (Fig. 4b). One possible interpretation for their selection is that Egyptian consumers are usually skeptical about new systems until they prove credibility. The same finding was identified in other contexts^51^, and further highlighted by the EC where demonstration projects were considered a main promotion strategy for NbS in different cities^23^.

#### Financial facilitations

Both groups highlighted the general need for financial facilitations (Fig. 4c), similar to other markets^51,98^. Even though having such facilitations was not associated with the acceptance of consumers in Egypt^69^ and in some countries^56,58^, the high selection of suppliers for this need is expected and can be attributed to the economic conditions in the country and initial costs of systems, in line with previous studies^46,47,63^. Despite the initial agreement on the need for facilitations, the results showed a limited ability of suppliers to convey the consumers’ priorities in this regard. Suppliers highly underestimated the clear preference of consumers for savings on existing expenses, such as getting reductions on taxes and house permit costs. On the contrary, they highly overestimated the preference for soft loans, which is regarded as a riskier option by consumers^69^ . The suppliers’ assumption that consumers would opt for getting funds or loans could be understood given the current practices, where reliance on lending options is escalating in Egypt^99,100^. However, we can conclude from our findings that consumers preferred to benefit from the cost-saving potential of BI-NbS without the hurdle of applying for loans, compared to the more costly products they purchase using lending options, such as cars and home appliances^100^. This preferred benefit corresponds to the socioeconomic development (SCh3) challenge outlined by IUCN^2,3^ (see *Introduction*).

#### Implementation aims

Looking into how respondents ranked the implementation aims of BI-NbS revealed some differences between the two groups (Fig. 4d). The suppliers successfully captured the two most important aims for consumers, however, for them, the social aim of enjoying the aesthetic view of greenery (SCh3&4) precedes the production aim of producing healthy edible plants (SCh4&5). In general, suppliers tended to overestimate the importance of social aims for consumers, such as gardening in leisure time and using systems to maintain social status (SCh3). Even though such social dimension is usually well appraised by suppliers and consumers in different contexts^46,48,58,101^, yet in this context, the selected social aims by consumers were not as diverse (Fig. 4d). This points to contextual differences in the perception of how BI-NbS can resolve social challenges. Nevertheless, the three top selections of consumers indicated a high appreciation of the benefits for physical and mental health (SCh4), also determined in related studies in the US and Portugal^38,60^. The suppliers overestimated the weight of the economic aim of reducing household food expenses (SCh4), which was not reflected in the consumers’ choices. This discrepancy may hint that consumers misunderstand the actual contribution of such farms to the households’ dietary needs^33,91^ and expenses reduction^79,89^. It also stresses the need for effective communication of BI-NbS cost-benefit analysis in the local context, as highlighted in other contexts as well^38,53^.

#### Production preferences

The preferred types of production out of BI-NbS were almost consistent across both surveys (Fig. 4e). The suppliers successfully portrayed the high preference for productive plants by consumers. A similar preference was highlighted in a study on productive façades in Singapore^102^ but was slightly overlooked in studies on green roofs and façades in Ghana and Iran^27,82^. A major difference between both groups was the lack of differentiation between decorative and shading plants in the suppliers’ responses compared to the higher appreciation of decorative plants in the consumers’ responses. One possible reason is the diverse benefits of decorative plants, in addition to their easier integration within envelope elements, compared to the spatial limitations of planting shading plants, which usually require deep planter boxes or (wooden) structures to climb on^103^.

#### Operation preferences

Similarly, the operation preferences depicted by suppliers and consumers were mostly consistent starting by self-operating the systems, followed by relying on support from individuals, i.e., hired caretakers or gardeners (Fig. 4f). However, suppliers overestimated the option of involving supplying companies in the operation of BI-NbS. This indicates the consumers’ higher appreciation of independent system operation where for them, hiring an individual is not the same as getting the suppliers’ support service, probably regarded as more costly, hence its lower selection. We hardly found any studies focusing on this detailed aspect of system operation, only one study touched upon the preferred follow-up time and watering system for operating a vertical farm in Singapore^102^. The limited discussion in the literature is likely attributed to the multiple operation modes that could differ depending on the contextual dynamics.

#### Implementation barriers

Overall, there were differences in the perceptions of both groups regarding the implementation barriers facing BI-NbS (Fig. 4g), such as the high underestimation of suppliers for how worrying the incurred time and effort in operation were for consumers. The suppliers’ lower selection might arise from their technical knowledge, being familiar with high-tech systems that are less effort and time-intensive. Moreover, suppliers understated the importance of running costs, the main barrier for consumers, probably for being biased toward system marketing. In fact, running costs were also a larger barrier than initial costs in studies on Asian and European cities^53,56,58^. Conversely, suppliers overstated the impact of high initial costs on consumers, as shown by other studies on European and American cities^95,101^. Again, such confusion about BI-NbS finances stresses the importance of effective communication of costs and savings by suppliers to consumers. Lastly, suppliers overrated the status quo bias of consumers, i.e., their fear of trying something new, which links to how crucial demonstration examples might be for the promotion of BI-NbS^23^ (see *Implementation conditions*). In general, our results highlighted multiple misconceptions and gaps between both groups about BI-NbS realities.

### Perspectives for BI-NbS market

Having identified the market trends as well as the differences and overlaps between the perceptions of suppliers and consumers in regard to BI-NbS following the EC recommendation to connect involved stakeholders^23^, we attempt to bridge these gaps by outlining recommendations for better systems uptake. First, to increase the sales of BI-NbS typologies, more marketing is needed for rooftop systems, while more technical development is needed for façade systems to ensure that their installation methods and costs are suitable for the context and attractive to the consumers. Second, to expand the market, suppliers could adopt a sector-wise community penetration approach using tailored marketing plans^104^ to diversify their market segments and target female consumers, gardening enthusiasts, and experienced gardeners, where such groups showed high system acceptance in the context^66^. Third, to achieve higher feasibility of commercially selling decorative/edible plants produced from BI-NbS, suppliers could promote the community-based management model of connecting small-scale individual farms to operate on a neighborhood scale. Such a model could provide reasonable produce for selling and securing profit for owners^91,105^, thus encouraging consumers to pursue economic gains from BI-NbS, which might eventually increase their implementation. In addition, it achieves a higher ecological value and positive impact on biodiversity, driven by the larger scale of BI-NbS implementation^3^. Fourth, suppliers need to shift from high-tech innovative hydroponics^35,88^, currently advocated for, to low-tech soil-based systems preferred by consumers for their lower costs and average productivity^66^. To establish a new market in a context like Egypt^35,106^, the focus should be directed first to affordable and acceptable BI-NbS for easier diffusion, and then it could be shifted to more sophisticated and profitable systems. As the market grows, competition increases, and prices decrease, encouraging consumers to adopt more innovative options to increase their production.

**Fig. 5:**
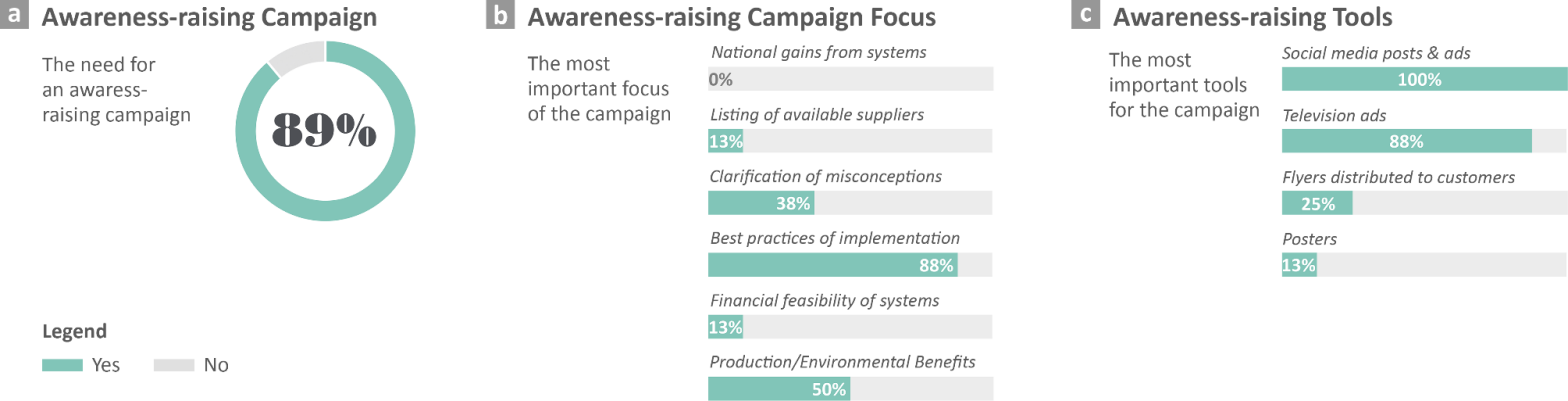
Results of the awareness campaign preferences. Suppliers (n = 15) were asked a yes/no question about the need for a campaign and were asked to select their most important focuses and tools for such a campaign.

Multiple perspectives were also derived from the supply–demand analysis of BI-NbS. First, suppliers need to focus their marketing strategies on addressing the anxieties and misconceptions of consumers, by showcasing successful projects of the relatively new BI-NbS using different channels (e.g. media, site visits, or exhibitions)^47,56,58^. Second, relentless efforts are needed to organize awareness-raising campaigns on the spatial requirements of available BI-NbS as well as their financial feasibility, for their highlighted positive influence on consumers’ uptake^61,63^. In more detail, in our survey, 89% of the surveyed suppliers confirmed the need for awareness raising, and 88% highlighted the importance of showcasing best practices (Fig. 5). Yet, the suppliers underrated the importance of clarifying the consumers’ misconceptions, selected by 38% and conveying the financial feasibility, selected by 13%. The importance of awareness raising for the MENA region, especially to clarify the BI-NbS financial feasibility and increase public knowledge of their potential, was highly stressed in a previous study^43^. Third, the diverse social benefits harnessed from the systems need more marketing in the context, while the environmental benefits, which proved contextual importance, need to be used as a selling point, especially since both types of benefits define the main societal challenges addressed by NbS in cities. Fourth, suppliers and policymakers need to advocate more for financial facilitations that ensure costs and tax reductions, and not for loans that are currently at the center of attention (e.g. green loans)^107^. Suppliers could also devise supporting financial mechanisms by facilitating payment via installment plans or via the “Buy Now Pay Later” BNPL model used for other products^100^. Fifth, technical knowledge transfer from the suppliers to consumers is necessary to facilitate the independent operation of systems, potentially through offering affordable workshops or training sessions^81^. In the end, the higher alignment of the supply and demand sides guarantees a higher BI-NbS market development and implementation in the context of Egypt and countries of the Global South characterized by the same climatic, socioeconomic, and market conditions.

### Study limitations

Despite the overall novelty of the study’s aim and findings, still there were some limitations. (1) The qualitative method and purposive sampling led to a sample size that is potentially limited in its generalizability, yet it was deemed suitable for the study design that investigated the perceptions of key market players. (2) Face-to-face surveys might have yielded more comprehensive results compared to online surveys^59^, but we used the latter due to COVID-19 regulations at the time of data collection. (3) In the market trends questions, suppliers reported estimates which might have led to some biases, as they were probably based on their expertise more than on factual data^50^. However, the questionnaire design proved successful in achieving the study aim that targeted a preliminary understanding of the market, which was mostly aligned with the general discourse in literature focused on Egypt as well as other countries.

## Conclusion

BI-NbS contribution to addressing societal challenges in urban areas will not have tangible manifestations until large-scale implementation takes place, especially in rapidly urbanizing countries of the Global South, such as Egypt. To pave the way for such implementation, it is necessary to identify the market conditions for BI-NbS and to scrutinize the gaps between key stakeholders shaping the market to ensure effective communication that promotes wider adoption. In reply to the first research question about BI-NbS market trends, our study revealed that the residential sector sales are still limited, thus supporting the cruciality of our analysis that investigated this sector. It concluded that the highest sales are achieved by rooftop systems, monopolized by high-income groups, and aimed at self-consumption of the produce and reduction of food expenses. In addition, high-tech hydroponics have the highest costs, feasibility, and productivity, while low-tech soil/sand-based systems have the lowest costs and productivity, with raised bed systems balancing low cost with average productivity. In reply to the second research question, we identified multiple gaps and overlaps between the perceptions of the suppliers and consumers regarding BI-NbS acceptance dynamics. Alignment between both groups is mostly evident in the results of the production and operation preferences as well as the anxieties and concerns about the systems, while it is partially evident in the results of the needed financial facilitations. Discrepancies between the two groups were clear in the results of the implementation conditions, aims, and barriers – highlighting the limited understanding of suppliers for what hinders or facilitates implementation from the consumers’ perspective. The study at hand attempted to reveal the instrumental role, currently overlooked by research, that could be played by suppliers in local markets, and the conditional link between the success in this role and the better understanding of consumers’ perceptions. The study outcomes could support suppliers and policymakers to successfully promote and implement the systems in the Egyptian market as well as in other emerging NbS markets in the Global South that share the same climatic, socioeconomic, and market conditions, and face similar societal challenges.

## Supplementary Information

Supplementary Information.

## Data Availability

The raw data supporting the conclusions of this article will be made available by the authors, without undue reservation.
