# Supplementary material for: Perceptions of building-integrated nature-based solutions by suppliers versus consumers in Egypt
Source: Sci Rep. 2024 Oct 30;14:26163. doi: 10.1038/s41598-024-76014-8 (PMC11525813; doi:10.1038/s41598-024-76014-8)
Supplement: Supplementary file 1 — Supplementary Information. [file 41598_2024_76014_MOESM1_ESM.docx]

**Supplementary Information**

**Perceptions of building-integrated nature-based solutions by suppliers versus consumers in Egypt**

# Mai A. Marzouk1,2, *, Mohamed A. Salheen3,4, and Leonie K. Fischer1

1Institute of Landscape Planning and Ecology ILPÖ, Faculty of Architecture and Urban Planning, University of Stuttgart, Stuttgart, Germany

2Department of Architecture, Faculty of Engineering, Ain Shams University, Cairo, Egypt

3Department of Urban Design and Planning, Faculty of Engineering, Ain Shams University, Cairo, Egypt

4Integrated Urbanism and Sustainable Design (IUSD) Program, Ain Shams University, Cairo, Egypt

*** Correspondence:**

Mai A. Marzouk: [mai-adel-fathy.marzouk@ilpoe.uni-stuttgart.de](mailto:mai-adel-fathy.marzouk@ilpoe.uni-stuttgart.de)

**Article Citation Reference:**

Marzouk, M. A., Salheen, M. A. & Fischer, L. K. Perceptions of building-integrated nature-based solutions by suppliers versus consumers in Egypt. Sci. Rep. (2024). <https://doi.org/10.1038/s41598-024-76014-8>

Supplementary Information

Supplementary Tables S1 and Table S2 introduce the original questions and response items of the Suppliers and Consumers surveys, which were analyzed in the article. The article focused specifically on Building-Integrated Nature-based Solutions (BI-NbS), implemented on building envelopes (rooftops, façades, and balconies) to produce decorative and edible plants. However, when developing both surveys and since the term “BI-NbS” is not commonly used in the context, especially among the suppliers and consumers, the term “Agricultural systems” was alternatively used to correspond to the commonly used Arabic term, which described the systems in the translated versions of the surveys. “Agricultural systems” had a clearer connotation to the systems and is understood as covering both the “Greening” and “Agricultural” options. The term was also clearly defined in the English and Arabic versions of the surveys so that the respondents understand what the term refers to and what systems and plant types it encompasses.

Supplementary Table S1: Consumers survey questions, response items, number, and percentage of respondents of each item. Questions about the social acceptance dynamics and sociocultural aspects are shown. n: number of respondents (n=274).

| **Variable** | **Original survey question** | **Original Response items** | **Respondents** | |
| --- | --- | --- | --- | --- |
|  |  |  | **n (#)** | **n (%)** |
| **Social Acceptance Dynamics** | | | | |
| **Anxiety about Systems** | What are the 4 most important worries about implementing agricultural systems? | Adding extra load on the building's structure | 55 | 20% |
|  |  | Risk of water leakage from the system | 164 | 60% |
|  |  | Attracting unwanted insects to the house | 176 | 64% |
|  |  | Plants getting eaten/damaged by birds/pests | 119 | 44% |
|  |  | Being new to the market & not fitting our buildings | 32 | 12% |
|  |  | Losing plants due to lack of knowledge/skills | 157 | 58% |
| **Implementation Conditions** | What are the 4 most important conditions to you for implementing agricultural systems? | House has sufficient area & needed utilities | 217 | 79% |
|  |  | Suppliers/technicians are available to assist | 164 | 60% |
|  |  | Systems are easily available in the market | 154 | 56% |
|  |  | Financial incentives are devised by the government | 64 | 23% |
|  |  | Building codes/regulations support implementation | 79 | 29% |
|  |  | A need to reduce pollution and improve environment | 164 | 60% |
|  |  | Have successful examples of system implementation | 135 | 49% |
|  |  | Specialists (engineers/architects) recommend systems | 90 | 33% |
|  |  | People important to me expect me to buy the systems | 17 | 6% |
| **Financial Facilitations** | What are the 2 most needed financial incentives, if any, to implement agricultural systems? | Tax reductions/rebates | 121 | 44% |
|  |  | Soft loans (reduced interest rates) | 36 | 13% |
|  |  | Investment grants (funds) | 52 | 19% |
|  |  | Reduction in house permit costs | 96 | 35% |
|  |  | Reduction in utilities costs | 180 | 66% |
|  |  | No financial incentives are needed | 26 | 10% |
| **Implementation Aims** | What is your most important aim for installing/reinstalling the agricultural system? (Please select 1 aim only) | Producing healthy edible plants for the household | 82 | 42% |
|  |  | Planting decorative or shading plants | 19 | 10% |
|  |  | Reducing the household's food expenses | 6 | 3% |
|  |  | Having a commercial activity by selling the produce | 7 | 4% |
|  |  | Increasing the house property value/resale potential | 0 | 0% |
|  |  | Maintaining a social status among peers | 1 | 0.4% |
|  |  | Providing an aesthetic view of greenery | 52 | 27% |
|  |  | Practicing an activity in leisure time | 3 | 2% |
|  |  | Strengthening relations with neighboring residents | 2 | 1% |
|  |  | Enjoying cleaner air and shade from plants | 44 | 23% |
| **Production Preferences** | What type of plants would you grow?  (You can select multiple options) | Productive plants (vegetables, fruits, herbs, etc.) | 150 | 78% |
|  |  | Decorative plants (flowers, Succulents, foliage, etc.) | 137 | 71% |
|  |  | Plants for shading (Jasmine, Bougainvillea, etc.) | 114 | 59% |
| **Operation Preferences** | What are your plans for the agricultural system operation/follow-up? (You can select multiple options) | Operate it on their own | 148 | 77% |
|  |  | Get support from individuals (caretaker, gardener, etc.) | 84 | 44% |
|  |  | Rely on supplier's operation/follow-up service | 46 | 24% |
| **Implementation**  **Barriers** | What are the 4 most important barriers to installing/reinstalling the agricultural system? | High demand of time and effort for operation/follow-up | 48 | 60% |
|  |  | High demand of knowledge for operation/follow-up | 42 | 53% |
|  |  | High initial costs of the system | 47 | 59% |
|  |  | High maintenance and running costs | 54 | 68% |
|  |  | High expenses compared to potential savings | 44 | 55% |
|  |  | Lack of trust in the system quality | 21 | 26% |
|  |  | Lack of trust in the implementing companies | 22 | 28% |
|  |  | Fear of trying something new (status-quo bias) | 23 | 29% |
| **Sociocultural Aspects** | | | | |
| **Age** | What is your age? | Under 20 | 1 | 0.4% |
|  |  | 20 to 40 | 174 | 64% |
|  |  | 41 to 60 | 77 | 28% |
|  |  | Above 60 | 22 | 8% |
| **Gender** | What is your gender? | Female | 149 | 54% |
|  |  | Male | 125 | 46% |
| **Educational Level** | What is your highest educational level? | No school completed | 0 | 0% |
|  |  | Secondary Education or equivalent | 9 | 3% |
|  |  | Bachelor's Degree or equivalent | 153 | 56% |
|  |  | Post Graduate Degree (M.Sc. or Ph.D.) | 112 | 41% |
| **Employment Status** | What is your current employment Status? | Full-time employed | 168 | 61% |
|  |  | Part-time employed | 22 | 8% |
|  |  | Self-Employed (freelance or business owner) | 28 | 10% |
|  |  | Not employed | 15 | 6% |
|  |  | Retired | 14 | 5% |
|  |  | Student | 12 | 4% |
|  |  | Volunteer | 0 | 0% |
|  |  | Stay-at-home mom/dad | 15 | 5% |
|  |  | Other | 9 | 3% |
| **Household monthly income** | What is your average monthly household income? (Optional) | < 5000 EGP | 35 | 16% |
|  |  | 5000 - 15000 EGP | 99 | 44% |
|  |  | 15000 - 30000 EGP | 50 | 22% |
|  |  | > 30000 EGP | 39 | 18% |
| **New city Location** | In which new city do you live? | 6th of October city | 24 | 9% |
|  |  | 15th of May city | 3 | 1% |
|  |  | 10th of Ramadan city | 4 | 2% |
|  |  | New Cairo city | 139 | 51% |
|  |  | Badr city | 5 | 2% |
|  |  | Shorouk city | 30 | 11% |
|  |  | Obour city | 13 | 5% |
|  |  | Sheikh Zayed city | 18 | 7% |
|  |  | Madinaty City | 5 | 2% |
|  |  | Rehab City | 1 | 0.4% |
|  |  | Future City | 1 | 0.4% |
|  |  | New Heliopolis City | 16 | 6% |
|  |  | New Administrative Capital | 0 | 0% |
|  |  | South of New Cairo City | 5 | 2% |
|  |  | New 6th of October City | 1 | 0.4% |
|  |  | October Gardens City | 2 | 1% |
|  |  | Extension of Sheikh Zayed | 0 | 0% |
|  |  | New Sphinx City | 0 | 0% |
|  |  | New Al-Warraq City | 1 | 0.4% |
|  |  | New El-Obour City | 5 | 2% |
|  |  | Mostakbal City | 2 | 1% |
| **Residence Type** | What is the type of your residence? | Flat in Apartment Building | 183 | 66% |
|  |  | Twin-house | 26 | 10% |
|  |  | Stand-alone villa | 55 | 20% |
|  |  | Other | 10 | 4% |
| **Residence Ownership** | What is the ownership of your residence? | Rented | 32 | 12% |
|  |  | Owned | 242 | 88% |
| **Area Restrictions on the Systems** | In your residential area, are there restrictions that prohibit installing the systems on the building's roofs, facades, or balconies? | Yes | 41 | 15% |
|  |  | No | 92 | 34% |
|  |  | Not sure | 141 | 51% |
| **Access to Shared Facilities** | In your flat, can you use the common/shared functions/facilities (e.g., Rooftop, garden, etc.)? | Yes | 110 | 61% |
|  |  | No | 59 | 32% |
|  |  | Other | 13 | 7% |

Supplementary Table S2: Suppliers survey questions, response items, number, and percentage of respondents of each item. Questions about the market trends and social acceptance dynamics are shown. n: number of respondents (n=15).

| **Variable** | **Original survey question** | **Original Response items** | **Respondents** | |  |
| --- | --- | --- | --- | --- | --- |
|  |  |  | **n (#)** | **n (%)** |  |
| **Market Trends** | | | | | |
| **Residential Sector Sales** | What is the percent of agricultural systems’ sales to residential buildings, out of your company’s total sales? | 0% | 1 | 11% |  |
|  |  | 25% | 5 | 56% |  |
|  |  | 50% | 2 | 22% |  |
|  |  | 75% | 1 | 11% |  |
|  |  | 100% | 0 | 0% |  |
| **BI-NbS Typologies Sales** | Where, in the house, are most of the sold agricultural systems installed? | On the rooftop | 9 | 75% |  |
|  |  | On the exterior wall/façade | 0 | 0% |  |
|  |  | In the balcony | 4 | 33% |  |
|  |  | Indoors | 0 | 0% |  |
| **Market Segments** | In the market, which income groups have a high rate of agricultural systems’ implementation? | Low-income groups | 2 | 22% |  |
|  |  | Middle-income groups | 4 | 44% |  |
|  |  | High-income groups | 6 | 67% |  |
|  | In the market, which income groups only have a high interest in the systems but without implementation? | Low-income groups | 3 | 33% |  |
|  |  | Middle-income groups | 6 | 67% |  |
|  |  | High-income groups | 7 | 78% |  |
| **Feasibility of BI-NbS Aims** | In your opinion, are the following production aims financially feasible for customers? | Edible plants production for self-consumption (yes) | 10 | 83% |  |
|  |  | Edible plants production for expenses reduction (yes) | 10 | 83% |  |
|  |  | Edible plants production for selling (yes) | 4 | 33% |  |
|  |  | Decorative/shading plants production for selling (yes) | 6 | 50% |  |
| **BI-NbS Costs and Productivity** | Which agricultural systems fall under the below categories? | |  |  |  |
|  | *Highest initial cost* | Extensive rooftop | 1 | 9% |  |
|  |  | Intensive rooftop | 1 | 9% |  |
|  |  | DWC Hydroponic Systems | 4 | 36% |  |
|  |  | NFT Hydroponic Systems | 6 | 55% |  |
|  |  | Aquaponic Systems | 2 | 18% |  |
|  |  | Sandponic Systems | 1 | 9% |  |
|  | *Lowest initial cost* | Raised beds/different containers | 3 | 30% |  |
|  |  | DWC Hydroponic Systems | 1 | 10% |  |
|  |  | Sandponic Systems | 2 | 20% |  |
|  |  | Planter Boxes and Pots | 4 | 40% |  |
|  | *Highest productivity* | Raised beds/different containers | 1 | 9% |  |
|  |  | DWC Hydroponic Systems | 3 | 27% |  |
|  |  | NFT Hydroponic Systems | 4 | 36% |  |
|  |  | Aeroponic Systems | 1 | 9% |  |
|  |  | Aquaponic Systems | 1 | 9% |  |
|  |  | Sandponic Systems | 1 | 9% |  |
|  |  | Integrated Rooftop Greenhouse | 1 | 9% |  |
|  | *Lowest productivity* | DWC Hydroponic Systems | 1 | 10% |  |
|  |  | NFT Hydroponic Systems | 1 | 10% |  |
|  |  | Sandponic Systems | 2 | 20% |  |
|  |  | Green Façade | 1 | 10% |  |
|  |  | Planter Boxes and Pots | 5 | 50% |  |
|  | *Highest feasibility (balancing costs & savings)* | Extensive rooftop | 1 | 10% |  |
|  |  | Intensive rooftop | 1 | 10% |  |
|  |  | Raised beds/different containers | 1 | 10% |  |
|  |  | DWC Hydroponic Systems | 3 | 30% |  |
|  |  | NFT Hydroponic Systems | 3 | 30% |  |
|  |  | Aquaponic Systems | 1 | 10% |  |
|  |  | Sandponic Systems | 1 | 10% |  |
|  |  | Planter Boxes and Pots | 1 | 10% |  |
| **Social Acceptance Dynamics** | |  |  |  |  |
| **Anxiety about Systems** | In your opinion, what are the 3 most important worries/concerns customers have about the agricultural systems? | Adding extra load on the building's structure | 4 | 33% |  |
|  |  | Risk of water leakage from the system | 7 | 58% |  |
|  |  | Attracting unwanted insects to the house | 5 | 42% |  |
|  |  | Plants getting eaten/damaged by birds/pests | 5 | 42% |  |
|  |  | Being new to the market & not fitting our buildings | 2 | 17% |  |
|  |  | Losing plants due to lack of knowledge/skills | 2 | 17% |  |
| **Implementation Conditions** | What are the 4 most important conditions that support implementation for customers? | House has sufficient area & needed utilities | 6 | 50% |  |
|  |  | Suppliers/technicians are available to assist | 7 | 58% |  |
|  |  | Systems are easily available in the market | 3 | 25% |  |
|  |  | Financial incentives are devised by the government | 5 | 42% |  |
|  |  | Building codes/regulations support implementation | 0 | 0% |  |
|  |  | A need to reduce pollution and improve environment | 2 | 17% |  |
|  |  | Have successful examples of system implementation | 9 | 75% |  |
|  |  | Specialists (engineers/architects) recommend systems | 3 | 25% |  |
|  |  | Peer pressure to buy the systems as a social status | 0 | 0% |  |
| **Financial Facilitations** | What are the 2 most needed financial incentives, if any, to support the implementation of agricultural systems? | Tax reductions/rebates | 1 | 11% |  |
|  |  | Soft loans (reduced interest rates) | 3 | 33% |  |
|  |  | Investment grants (funds) | 1 | 11% |  |
|  |  | Reduction in house permit costs | 1 | 11% |  |
|  |  | Reduction in utilities costs | 4 | 44% |  |
|  |  | No financial incentives are needed | 1 | 11% |  |
| **Implementation Aims** | In your opinion, what are the 4 most important aims customers want to achieve when installing agricultural systems? | Producing healthy edible plants for self-consumption | 8 | 89% |  |
|  |  | Planting decorative or shading plants | 1 | 11% |  |
|  |  | Reducing the household's food expenses | 4 | 44% |  |
|  |  | Having a commercial activity by selling the produce | 0 | 0% |  |
|  |  | Increasing the house property value/resale potential | 0 | 0% |  |
|  |  | Maintaining a social status among peers | 2 | 22% |  |
|  |  | Providing an aesthetic view of greenery | 9 | 100% |  |
|  |  | Practicing an activity in leisure time | 6 | 67% |  |
|  |  | Strengthening relations with their neighbors | 0 | 0% |  |
|  |  | Enjoying cleaner air and shade from plants | 4 | 44% |  |
| **Production Preferences** | What type of plants would the customers prefer to grow in residential buildings?  (You can select multiple options) | Productive plants (vegetables, fruits, herbs, etc.) | 6 | 50% |  |
|  |  | Decorative plants (flowers, Succulents, etc.) | 3 | 25% |  |
|  |  | Plants for shading (Jasmine, Bougainvillea, etc.) | 3 | 25% |  |
| **Operation Preferences** | Which option do customers prefer for the agricultural systems’ operation/follow-up? (You can select multiple options) | Operate it on their own | 9 | 75% |  |
|  |  | Get support from individuals (caretaker, gardener, etc.) | 6 | 50% |  |
|  |  | Rely on supplier's operation/follow-up service | 6 | 50% |  |
| **Implementation Barriers** | In your opinion, what are the 4 most important barriers that hinder customers from installing the agricultural systems? | High demand of time and effort for operation/follow-up | 2 | 22% |  |
|  |  | High demand of knowledge for operation/follow-up | 5 | 56% |  |
|  |  | High initial costs of the system | 7 | 78% |  |
|  |  | High maintenance and running costs | 4 | 44% |  |
|  |  | High expenses compared to potential savings | 4 | 44% |  |
|  |  | Lack of trust in the system quality | 5 | 56% |  |
|  |  | Lack of trust in the implementing companies | 3 | 33% |  |
|  |  | Fear of trying something new (status-quo bias) | 4 | 44% |  |
